# Role of oligandrin in enhancing post-harvest disease resistance in cherry tomato through salicylic acid and jasmonic acid signaling pathways

**DOI:** 10.1128/aem.00421-25

**Published:** 2025-06-13

**Authors:** Jia-hao Sun, Jia-hui Xu, Chuanzhi Kang, Lu Cheng, Yu-tang Gao, Feng-qiao Hu, Jian Liang, Lan-Ping Guo, Xiao-kui Ma

**Affiliations:** 1 Key Laboratory of Medicinal Resources and Natural Pharmaceutical Chemistry, Ministry of Education, National Engineering Laboratory for Resource Developing of Endangered Chinese Crude Drugs in Northwest of China, College of Life Science, Shaanxi Normal University 162758, Xi'an, Shaanxi, China; 2 State Key Laboratory for Quality Ensurance and Sustainable Use of Dao-di Herbs, National Resource Center for Chinese Materia Medical, China Academy of Chinese Medical Sciences 666361 , Beijing, China; 3 Yanchi County Science and Technology Bureau, Ningxia, China; Anses, Maisons-Alfort Laboratory for Food Safety, Maisons-Alfort, France

**Keywords:** oligandrin, induced disease resistance, gene expression, defense-related enzyme activities, *Alternaria nees*, *Pythium oligandrum*, post-harvest preservation

## Abstract

Salicylic acid (SA) and jasmonic acid (JA) pathways are crucial components of plant defense mechanisms; however, their roles in post-harvest preservation remain inadequately explored. Cherry tomatoes are highly susceptible to fungal pathogens, such as *Alternaria nees*, after harvest, leading to significant post-harvest losses. This study investigates the effects of oligandrin, an elicitin protein derived from *Pythium oligandrum*, on enhancing the resistance of harvested cherry tomatoes to *A. nees*. Oligandrin treatment significantly reduced disease symptoms, including black spots and decay, during storage without affecting *A. nees* spore germination. Treated samples exhibited enhanced activities of defense-related enzymes, including polyphenol oxidase (PPO), peroxidase (POD), phenylalanine ammonia-lyase (PAL), and catalase (CAT), compared to controls. Key SA-responsive genes, such as *NPR1* (Non-expressor of Pathogenesis-Related Genes 1), *TGA2* (TGACG-Binding Factor 2), *WRKY70*, and *PR1* (Pathogenesis-Related Protein 1), were upregulated within 48 h, indicating activation of the SA signaling pathway. Additionally, *JAZ1* (Jasmonate ZIM-Domain 1), a negative regulator of JA signaling, was elevated at both 24 and 48 h, while *MYC2* (Myelocytomatosis 2), a transcription factor involved in the JA pathway, showed significant upregulation, suggesting activation of the JA signaling pathway. These findings demonstrate that oligandrin enhances post-harvest fungal disease resistance by modulating both SA and JA pathways and sustaining elevated defense enzyme activity, offering a promising strategy for improving post-harvest preservation and quality maintenance of cherry tomatoes.

**IMPORTANCE:**

This study provides compelling evidence that oligandrin activates both salicylic acid and jasmonic acid signaling pathways in cherry tomatoes, enhancing resistance to *A. nees*. This dual activation not only deepens our understanding of oligandrin’s mechanisms but also underscores the potential of oligandrin and its producer *P. oligandrum* as a biocontrol agent for post-harvest preservation. By modulating plant immunity and promoting defense enzyme activity, oligandrin strengthens resistance to fungal diseases, offering a promising strategy for maintaining the quality and extending the shelf life of berry-like crops. Given the successful use of *P. oligandrum* as a biopesticide in Europe and North America, oligandrin and its producer *P. oligandrum* present an environmentally friendly alternative to chemical pesticides for post-harvest disease management. This research lays the groundwork for further studies aimed at optimizing the application of *P. oligandrum* in real-world agricultural settings, establishing it as a reliable, sustainable tool for both agricultural and post-harvest management practices. These findings contribute to advancing biocontrol strategies for safer, more sustainable food production systems.

## INTRODUCTION

Current public health concerns and stringent regulatory policies have significantly restricted the use of chemical agents for controlling post-harvest fungal rot in agricultural products. In response, biological control agents (BCAs), such as *Pythium oligandrum*, have emerged as promising alternatives, gaining considerable attention in Europe and North America. *P. oligandrum* not only directly protects plants from fungal pathogens but also activates plant defense mechanisms, enhancing plant growth and overall vigor (1). The protective efficacy of *P. oligandrum* arises from synergistic mechanisms, including antagonism against soil-borne diseases, secretion of auxin precursors that promote plant growth, and microbiome-associated molecular patterns, such as oligandrin, that stimulate plant defense responses (2).

Oligandrin, a key extracellular protein of approximately 10 kDa derived from *P. oligandrum* cultures, plays a critical role in enhancing plant resistance to disease (3). This bioactive protein has been shown to trigger resistance in tomatoes against root rot and in grapes against *Botrytis cinerea*, stimulating plant defense responses against pathogens (4, 5). When grape leaves were treated with oligandrin, phenolic compound accumulation and cell wall adhesion were observed without triggering a hypersensitive response (HR) (4). Additionally, oligandrin-mediated resistance against *B. cinerea* is facilitated through the activation of the jasmonic acid (JA) signaling pathway (6). However, research on the application of *P. oligandrum* and oligandrin in post-harvest preservation or mold control remains limited, hindering the widespread use of these biocontrol agents in postharvest applications.

Cherry tomatoes (*Lycopersicon esculentum*) are particularly vulnerable to fungal pathogens, such as *Alternaria nees*, due to their high water content and nutrient-rich composition (7–9). *A. nees* is a significant contributor to post-harvest diseases in tomatoes, leading to substantial economic losses during storage and transportation. Infected mature cherry tomatoes typically exhibit black-brown lesions, which start at fungal penetration sites and manifest as oval or circular, slightly sunken brown spots often accompanied by black mildew (10). The progression of these lesions can render the fruit unmarketable, further exacerbating losses during post-harvest handling. Therefore, reducing post-harvest fungal rot and inhibiting fungal diseases are among the greatest challenges currently faced in preserving cherry tomatoes and other berry-like crops after harvest.

To mitigate post-harvest fungal rot, various strategies have been explored, including the use of natural approaches, such as melatonin (11), *Cryptococcus laurentii* (12), L-glutamate, and yeast cell wall chitin. Among these, the integration of biocontrol agents, like *P. oligandrum*, offers a promising and innovative strategy for enhancing resistance in cherry tomatoes. By strengthening the fruit’s inherent resistance, *P. oligandrum* may effectively combat fungal pathogens and enhance fruit health, providing an alternative to synthetic fungicides for post-harvest preservation management.

Oligandrin produced by *P. oligandrum* has been shown to activate the ethylene-dependent signaling pathway, inducing resistance to *B. cinerea* and reducing the severity of gray mold disease (5). Additionally, previous studies have highlighted oligandrin’s role in stimulating immune responses in tomatoes primarily through the mediation of the JA signaling pathway (6). However, there is limited research on the effectiveness of *P. oligandrum* or oligandrin in post-harvest preservation, particularly in enhancing resistance against *A. nees* in cherry tomatoes. Investigating the systemic resistance of cherry tomatoes to *A. nees* could provide valuable insights into post-harvest preservation strategies and serve as a model for similar crops.

The activation of systemic immunity in plants, whether through local plant-microbe interactions or active ingredients, has been a central focus in the study of systemic acquired resistance (SAR) and induced systemic resistance. These immune responses are regulated by phytohormones, including salicylic acid (SA), jasmonic acid (JA), and ethylene. Notably, pre-treatment with elicitors like oligandrin can prime plants, enhancing their defense mechanisms and providing heightened protection against subsequent pathogen attacks (3). JA and SA signaling pathways not only contribute to pathogen defense but also influence various aspects of plant development, reproduction, and stress responses (13). Induced resistance triggered by biocontrol agents like oligandrin offers broad-spectrum protection against fungal, bacterial, and viral infections (14), providing sustained defense against a wide range of pathogens.

Despite decades of research on these signaling pathways, the role of induced systemic resistance in post-harvest preservation remains poorly understood. The application of biocontrol agents like oligandrin and *P. oligandrum* to post-harvest crops, such as cherry tomatoes, has not been fully explored. Measuring the activity of defense hormone-responsive genes is critical for understanding immune signaling and assessing the effectiveness of these biocontrol approaches (15).

In this study, we investigate the role of oligandrin derived from *P. oligandrum* in enhancing systemic resistance in cherry tomatoes against *A. nees* during storage. We focused on how oligandrin modulates the SA/JA signaling network, primes key defense enzymes, such as polyphenol oxidase and peroxidase, and influences the transcriptional reprogramming of NPR1- and MYC2-mediated responses. By elucidating these molecular mechanisms, this research aimed to address a critical gap in understanding how oligandrin or *P. oligandrum* activates plant defense systems at the molecular level. Our findings will contribute to developing *P. oligandrum*-based biocontrol solutions as viable alternatives to conventional chemical fungicides for post-harvest preservation, advancing more sustainable and effective strategies for controlling post-harvest diseases, such as fungal rot in cherry tomatoes and similar crops, with broader implications for agricultural practices and food security.

## MATERIALS AND METHODS

### Fruit pretreatment and storage conditions

The mature red cherry tomato fruits (*Lycopersicon esculentum* var. *cerasiforme*) were sourced from Zhu Que Agricultural and Sideline Product Logistics Center in Xi'an, Shaanxi Province, China. Intact fruits with uniform size and free from defects and decay were selected and then surface-sterilized in 0.1% (v/v) sodium hypochlorite aqueous solution for 2 min, rinsed thoroughly with tap water, and air-dried. The fruits were stored in enclosed plastic trays at 25°C with high relative humidity (90–95%) for further experiments.

### Oligandrin preparation

Oligandrin was extracted following previously documented procedures (3), which allowed the recovery of large amounts of oligandrin in a pure form from the culture filtrates of *P. oligandrum*. In brief, culture filtrates (5 L) were subjected to a 10-fold concentration through vacuum evaporation at 35°C. The resulting concentrated product underwent a 24 h dialysis process in deionized water at 4°C. To this, 15 mL of 0.34 M sodium acetate was introduced, adjusting the pH to 3.5 using 10% (v/v) aqueous trifluoroacetic acid. Subsequently, the solution underwent vacuumization at 35°C and was dissolved in a mixture of 40% acetonitrile and 50 mM sodium formate solution. The protein purity was confirmed to be homogeneity, as demonstrated by SDS-PAGE. The electrophoretic profile of the oligandrin after SDS-PAGE indicated a single-stained band with a molecular mass of approximately 10 kDa, which compared well with molecular mass markers (3). The concentration of oligandrin in solution was quantified following the BCA method.

### Oligandrin treatment and infection of *A. nees*

Cherry tomato fruits were disinfected by immersion in a 0.1% (v/v) sodium hypochlorite solution for 2 min, followed by rinsing with sterile distilled water (SDW). The fruits were then allowed to air-dry. To create uniform wounds, the fruit peels were punctured at the center of the equatorial diameter to a depth of 2 mm using a sterile 5 mm diameter borer.

To assess the effect of oligandrin on disease incidence following *A. nees* infection, oligandrin solutions (20 µL) at concentrations of 2, 5, 10, and 20 µM were applied to the wounds using a sterilized applicator. The control group was treated with an equal volume of SDW. After a 24 h incubation period at room temperature and humidity for equilibration, each fruit was inoculated with 20 µL of *A. nees* spore suspension (1 × 10⁵ spores/mL) at the wound site using a sterilized applicator. Control fruits received 20 µL of SDW. The fruits were then incubated in humid chambers (90–95% relative humidity) at 25 ± 2°C using polyethylene trays lined with moistened paper towels until symptom development occurred.

To determine the optimal duration for oligandrin treatment to reduce disease incidence caused by *A. nees*, the optimal oligandrin concentration identified previously was applied to the wound sites using a sterilized applicator. Control fruits received SDW. Inoculations were carried out at 24, 48, and 72 h after oligandrin treatment with 20 µL of *A. nees* spore suspension (1 × 10⁵ spores/mL) at the wound sites. Each treatment was performed in triplicate, with 30 fruits per replicate.

Disease incidence was recorded at 3, 4, and 5 days post-inoculation of *A. nees*. The disease incidence was calculated as the proportion of infected tomatoes per treatment group. Each treatment consisted of three replicates, with 30 fruits per replicate. The oligandrin concentration that resulted in the lowest disease incidence was identified as the optimal concentration for disease resistance induction, which was used in subsequent experiments.

The *A. nees* spore suspension was prepared following standard laboratory methods, and the concentration of the suspension was adjusted to 1 × 10⁵ spores/mL using a hemocytometer for further experiments.

To assess the effect of oligandrin on the expression of genes involved in JA and SA signaling, oligandrin treatments and controls were conducted as described above but without inoculation of the *A. nees* spore suspension. Fruit tissue samples (excluding the peel) were collected at 24 and 48 h post-treatment. The samples were immediately frozen in liquid nitrogen and stored at −80°C. Ten fruits were sampled per timepoint for each treatment group.

### Influence of different concentrations of oligandrin on spore germination of *A. nees in vitro*

The spore suspension of *A. nees* (1 × 10^5^ cells mL^−1^) was cultured in PDA solution containing oligandrin at different concentrations (2, 5, 10, and 20 µM) at 28°C and 160 rpm for 12 h. The spore germination in different cultures was observed under an optical microscope. The ratio of spore germination was determined by counting 50 spores.

### Assay of defense-related enzyme activity in cherry tomato fruits

For enzyme assays, fresh fruit tissue samples (excluding the peel) were collected 1 to 2 mm beneath the surface at different time points after treatment. Each sample was obtained from 10 fruits and stored in polypropylene tubes at −80°C until further processing. All procedures for enzyme extraction were conducted on ice at 4°C.

For enzyme activity analysis, 1 g of the frozen tissue was ground using a mortar and pestle with 4 mL of chilled 50 mM sodium phosphate buffer (pH 7.8), supplemented with 1.33 mM EDTA and 1% (w/v) polyvinylpyrrolidone. For phenylalanine ammonia-lyase (PAL) analysis, 1 g of frozen sample was blended with 3.6 mL of 200 mM sodium borate buffer (pH 8.8). To determine polyphenol oxidase (PPO) and catalase (CAT) activities, 2 g of tissue was ground with 10 mL of 100 mM sodium phosphate buffer (pH 6.4) containing 0.2 g of polyvinylpolypyrrolidone. After homogenization, the mixtures were centrifuged at 12,000 ×*g* at 4°C for 10 min. The supernatants were then collected for enzyme activity. Enzyme activity was expressed as units per milligram of protein.

Peroxidase (POD) activity was determined based on a previous method with slight modifications (16). The reaction mixture included 100 µL of crude enzyme extract, 140 µL of 0.3% (v/v) guaiacol, and 60 µL of 0.3% (v/v) H₂O₂. PPO activity was measured at 420 nm every 20 s for 3 min. One unit of POD activity was defined as the amount of enzyme that caused a 0.01 increase in absorbance at 470 nm per minute. CAT activity was assayed as described previously (17). The reaction mixture consisted of 250 µL of 30 mM H_2_O_2_ and 50 µL of crude enzyme extract. The reaction was detected immediately at 240 nm every 30 s for 5 min. One unit of CAT activity was defined as the amount of enzyme that produced a decrease of A_240_ by 0.01 per min. PPO activity was assayed based on the previous method (18). The reaction mixture consisted of 250 µL of 30 mM H₂O₂ and 50 µL of crude enzyme extract. The reaction was monitored at 240 nm every 30 s for 5 min. One unit of CAT activity was defined as the amount of enzyme that caused a 0.01 decrease in absorbance at 240 nm per minute. PPO activity was measured based on the previous method, with the reaction mixture consisting of 250 µL of 10 mM catechol and 50 µL of crude enzyme extract. The reaction was monitored at 398 nm every 30 s for 5 min. One unit of PPO activity was defined as the amount of enzyme that caused a 0.01 increase in absorbance at 398 nm per minute. PAL activity was analyzed as described previously (19). The reaction mixture included 500 µL of crude enzyme extract and 200 µL of 50 mM L-phenylalanine solution. The reaction was terminated by adding 40 µL of 6 M HCl and incubating at 37°C for 4 h. The reaction was monitored at 290 nm every hour for 5 h. One unit of PAL activity was defined as the amount of enzyme that caused a 0.01 increase in absorbance at 290 nm per hour (12).

### RNA extraction from cherry tomatoes and reverse-transcription quantitative PCR

Total RNA was extracted from cherry tomato tissue using a TRIzol-based protocol. Ground tissue was homogenized in 1 mL TRIzol reagent and incubated at room temperature for 5 min to lyse the cells. After adding 200 µL chloroform and centrifuging (12,000 × *g*, 10 min, 4°C), the aqueous phase containing RNA was collected. RNA was precipitated by adding 750 µL isopropanol, incubating on ice for 10 min, and centrifuging (12,000 × *g*, 10 min). The pellet was washed with 75% ethanol and air-dried. The RNA pellet was dissolved in RNase-free water and treated with DNase I to remove genomic DNA. RNA concentration was measured using a spectrophotometer, and RNA purity was confirmed with an A_260_/A_280_ ratio of 1.8–2.0. RNA integrity was further assessed by gel electrophoresis.

Total RNA was reverse-transcribed into complementary DNA (cDNA) using the Evo M-MLV Reverse-Transcription Premix Kit (TaKaRa, China) with a PCR amplification apparatus (BIOER, China). The reaction was carried out in a 20 µL volume at 37°C for 15 min, followed by a 5 s incubation at 85°C. The resulting cDNA was then used for quantitative PCR (qPCR) in a 20 µL reaction using SYBR Premix Ex Taq II (Tli RNaseH Plus) with a Real-Time PCR System (Light Cycler 96, USA).

The thermal cycling conditions were set as follows: initial denaturation at 95°C for 30 s, followed by 45 cycles of 5 s at 95°C and 30 s at 60°C. To ensure specificity of the amplification, a melting curve analysis was performed with the following program: 5 s at 95°C, 60 s at 60°C, 1 s at 95°C, and 30 s at 50°C.

Gene expression levels were calculated using the 2^−ΔΔCT^ method, with *actin* serving as the internal reference gene for normalization. The fold changes in expression were determined relative to the control group. To evaluate the transcriptional levels of genes associated with JA and SA signaling in cherry tomatoes, genes responsive to SA and JA were selected for analysis using qPCR. The gene-specific primers designed and purchased from Sangon Biotech (Shanghai, China) are listed in Table S1 at https://github.com/jiahaoSun-1205/AEM00421-25R1_Supplemental_material/blob/main/AEM00421-25R1_Supplemental_material.docx. Each qPCR reaction was performed in triplicate.

### Statistics

Data are presented as means ± standard error of mean. Statistical significance was determined using GraphPad Prism 8 by *t*-test. Asterisks (*) indicate significant differences evaluated for each time interval between oligandrin treatment and its control (*P ≤ 0.05, **P ≤ 0.01, ***P ≤ 0.001).

## RESULTS

### Impact of oligandrin treatment on the infection of *A. nees* in cherry tomatoes

**Fig 1:**
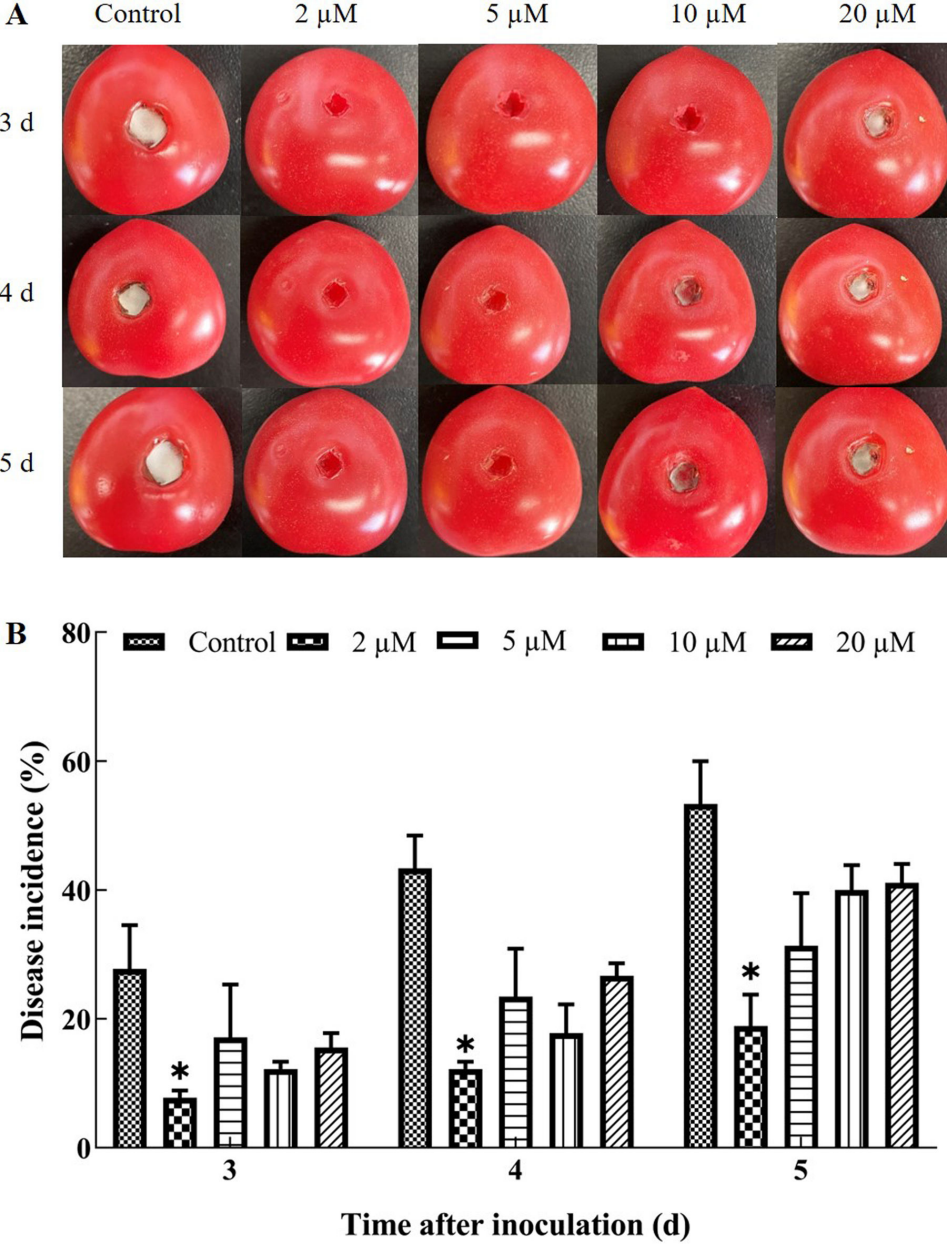
Effect of oligandrin treatment at different concentrations on disease incidence in cherry tomatoes infected with *Alternaria nees*. (**A**) Representative photographs of cherry tomatoes taken on days 3, 4, and 5 post-inoculation with *A. nees*. (**B**) Disease incidence in cherry tomatoes treated with oligandrin compared to the control group treated with sterile distilled water. Error bars represent the standard error of the mean from three independent replicates. *P* ≤ 0.05 indicates a significant difference compared to the control at each time point.

The resistance of cherry tomatoes to *A. nees* infection was assessed following oligandrin treatment. As shown in Fig. 1A, oligandrin treatments significantly reduced disease incidence compared to the control group. In the treated fruits, mycelial growth of *A. nees* was visibly inhibited, while white mycelia were observed in the control group, indicating *A. nees* growth. In Fig. 1B, the 2 µM oligandrin treatment exhibited the most substantial reduction in disease incidence (18.89% ± 4.84%) compared to the control group (53.33% ± 6.67 %). The 2 µM oligandrin treatment consistently reduced infection over the third, fourth, and fifth days post-inoculation (*P* < 0.05), establishing 2 µM as the optimal concentration for controlling disease incidence. Further increases in oligandrin concentration did not result in additional reductions in disease incidence, suggesting a saturation point for the protein’s interaction with plant cell surfaces. This is consistent with previous studies suggesting that once receptor sites were saturated, additional protein concentration does not enhance the effect (20).

### The influence of the duration of oligandrin treatment on the disease incidence in cherry tomatoes

**Fig 2:**
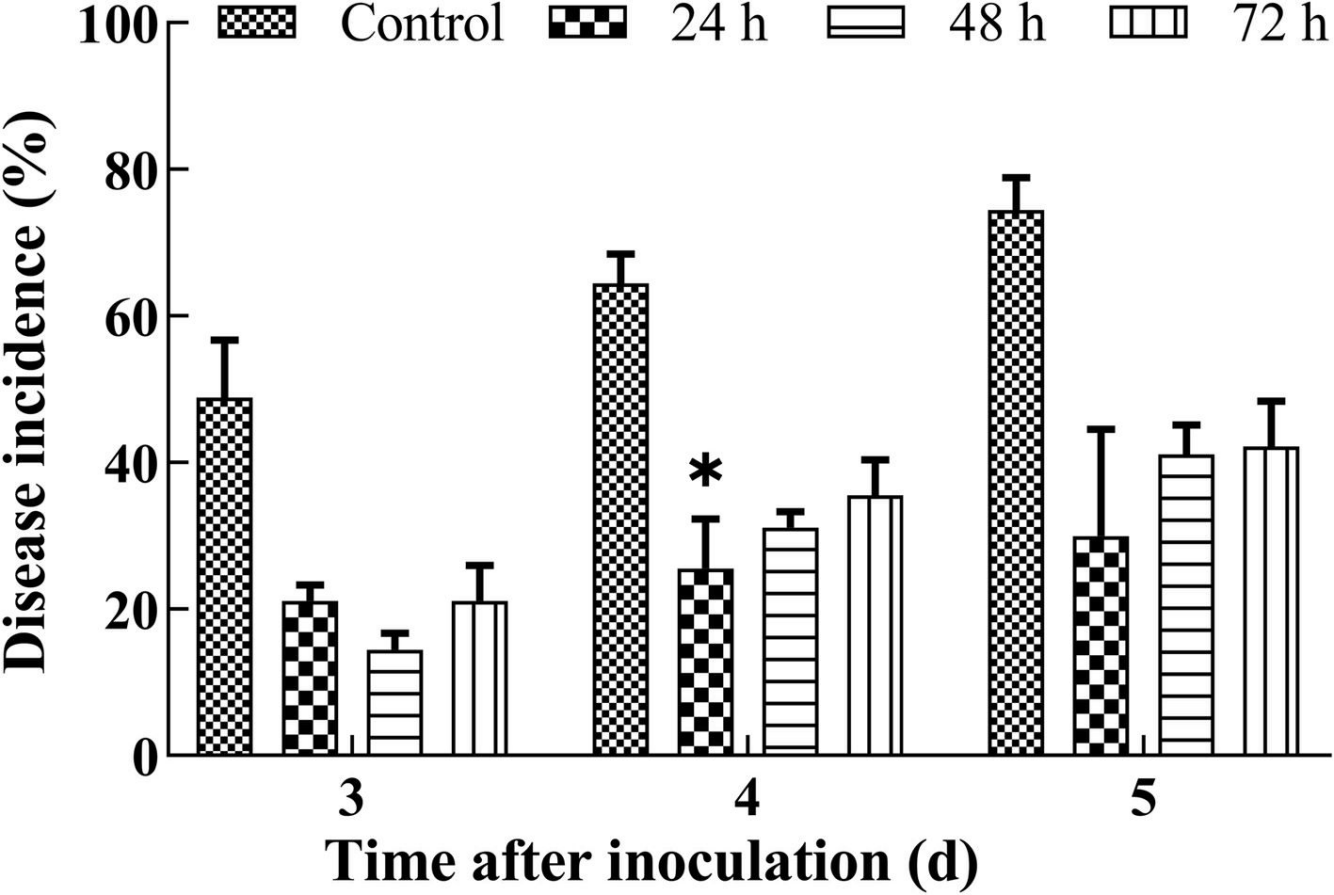
Effect of oligandrin treatment durations on disease incidence in cherry tomatoes infected with *A. nees*. Oligandrin treatments were applied for 0 (control), 24, 48, or 72 h. Disease incidence was measured on days 3, 4, and 5 following *A. nees* inoculation. Error bars represent the standard error of the mean from three independent replicates. *P* ≤ 0.05 indicates significant differences compared to the control at each time point.

As shown in Fig. 2, varying the duration of 2 µM oligandrin treatment significantly influenced disease incidence in cherry tomatoes infected with *A. nees* (*P* < 0.05). Disease incidence increased with time for all groups, but oligandrin treatment consistently reduced infection rates. The 24 h treatment exhibited the most significant protective effect, with a marked reduction in disease incidence on days 3, 4, and 5. Specifically, at day 5, the 24-hour treatment reduced disease incidence by approximately 44.44% compared to untreated controls.

Interestingly, extending the treatment duration to 48 or 72 h did not further reduce disease incidence. In fact, the efficacy of the 48 and 72 h treatments was similar and did not show any significant improvement over the 24 h treatment (day 3: 24 h vs. 48/72 h, *P* = 0.3). These findings suggest a critical window for oligandrin’s effect, where the 24 h treatment provides optimal protection, beyond which no additional benefit is gained from prolonged exposure.

The decline in the protective effect over time of the 24 h treatment (from 78.89% reduction on day 3 to 70.0% on day 5) further supports the hypothesis of membrane receptor saturation, where prolonged treatment may exceed the binding capacity of receptor sites, limiting the plant’s ability to enhance its defense response (6). This observation contrasts with traditional fungicides, which act based on concentration, emphasizing oligandrin’s unique mechanism of action potentially involving immune priming rather than direct toxicity.

### The *in vitro* effects of oligandrin treatment on spore germination of *A. nees*

The spore germination rate of *A. nees* was assessed under different concentrations of oligandrin (see Fig. S1 at https://github.com/jiahaoSun-1205/AEM00421-25R1_Supplemental_material/blob/main/AEM00421-25R1_Supplemental_material.docx). No significant difference in spore germination was observed between the oligandrin treatments and the control group (78.00% ± 5.83%), indicating that oligandrin did not directly inhibit the germination process of the pathogen. This finding is consistent with previous research on another elicitor protein, the cell wall protein from *Pythium oligandrum*, which similarly showed no interaction with the pathogen in terms of spore germination (21).

### Changes in defense-related enzyme activities induced by oligandrin

**Fig 3:**
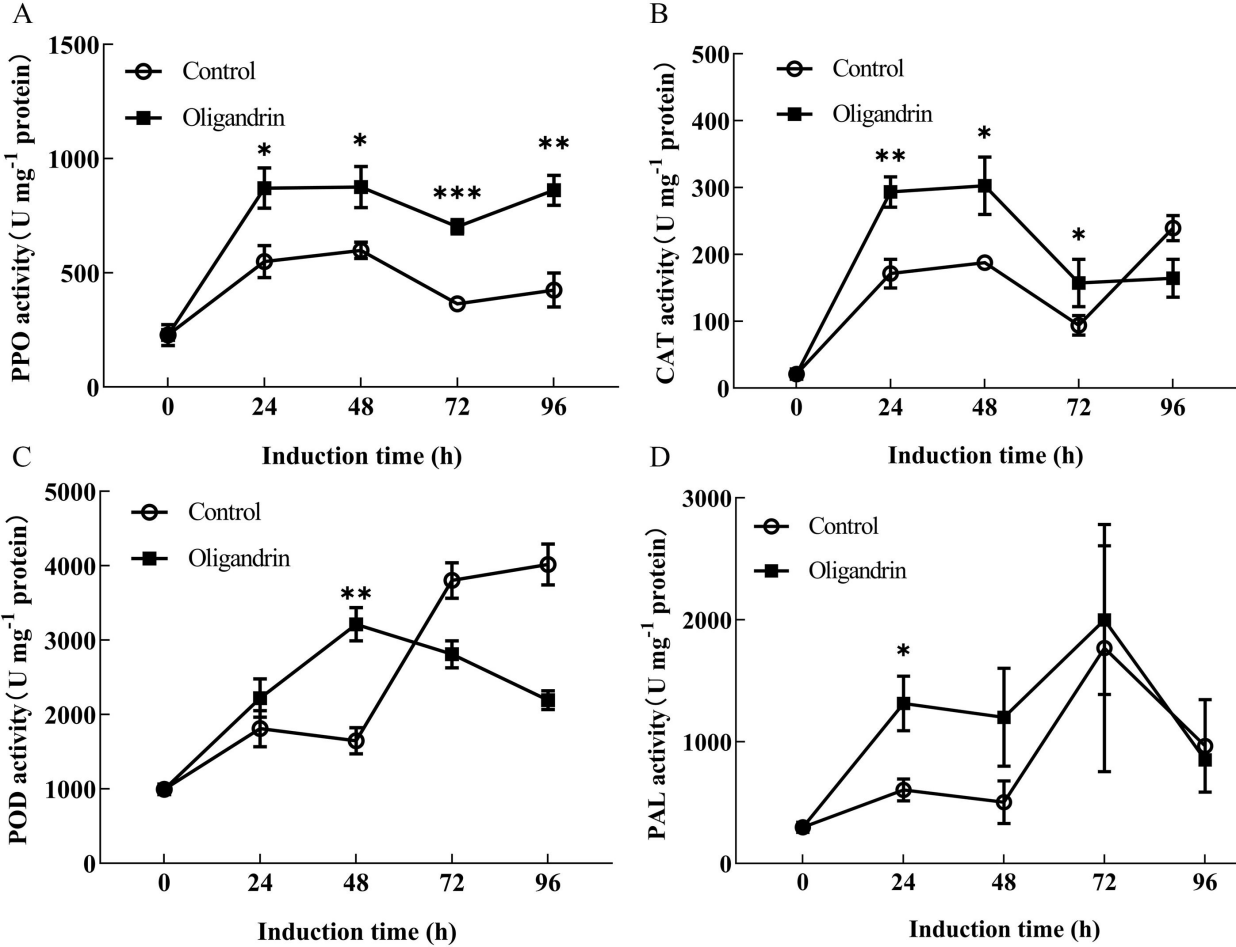
Effect of oligandrin treatment on the activities of (**A**) polyphenol oxidase (PPO), (**B**) catalase (CAT), (**C**) peroxidase (POD), and (**D**) phenylalanine ammonia-lyase (PAL) in cherry tomatoes. Error bars represent the standard error of the mean from three independent replicates. * P ≤ 0.05, ** P ≤ 0.01, and *** P *≤* 0.001 compared to the control at each time point.

The changes in defense-related enzyme activities induced by oligandrin treatment are shown in Fig. 3, where alterations in the activities of PPO, CAT, POD, and PAL are presented. These enzymes play crucial roles in plant defense, and their activities were significantly enhanced by oligandrin treatment, contributing to the increased disease resistance observed in cherry tomatoes.

Specifically, PPO activity increased progressively until 48 h, and then stabilized (Fig. 3A). The PPO activity was enhanced by 58.54%, 46.27%, 92.90%, and 102.76% at 24 h, 48 h, 72 h, and 96 h, respectively, compared to the control group (*P* < 0.05). This suggests that oligandrin treatment induces a sustained oxidative response, which may help in limiting pathogen growth and enhancing the overall defense mechanism of the plant. Similarly, CAT activity showed a consistent increase following oligandrin treatment (Fig. 3B), with notable increases of 71.23% at 24 h and 61.25% at 48 h compared to the control (*P* < 0.05). CAT is involved in the breakdown of reactive oxygen species (ROS), and its enhancement indicates that oligandrin helps the plant manage oxidative stress, which is an essential aspect of plant immune responses.

In contrast to PPO and CAT, POD activity exhibited a more dynamic response (Fig. 3C). After oligandrin treatment, POD activity increased rapidly and reached its peak at 48 h with a 95.18% increase compared to the control (*P* < 0.01). POD plays a critical role in strengthening cell walls and detoxifying ROS, and the rapid increase in its activity suggests that oligandrin primes the plant for an immediate defense response, particularly in cell wall fortification.

Moreover, PAL activity, which is involved in the biosynthesis of phenolic compounds, exhibited a distinct temporal pattern (Fig. 3D). The oligandrin treatment resulted in significantly higher PAL activity at 24, 48, and 72 h, with increases of 117.51, 138.79, and 13.01%, respectively, compared to the control (*P* < 0.05). This suggests that oligandrin may activate pathways leading to the synthesis of secondary metabolites, such as lignin and flavonoids, which contribute to the plant’s defense system.

These findings indicate that oligandrin treatment triggers a multifaceted defense response in cherry tomatoes, enhancing the activities of key enzymes involved in oxidative stress management and structural reinforcement. The progressive increase in PPO and CAT activities, followed by the peak in POD and PAL activities, highlights the temporal regulation of defense mechanisms. While PPO and CAT activities peak earlier, POD and PAL activities increase later, suggesting that oligandrin induces a coordinated and time-dependent defense response. PAL, a key enzyme in the phenylpropanoid pathway, indicates that oligandrin may also enhance the synthesis of lignin and phytoalexins, substances known to increase plant resistance to pathogens (22, 23). These enzymes are integral to oxidative stress responses and the reinforcement of cell wall integrity. In our study, the rapid enhancement of their activities following oligandrin treatment suggests that oligandrin may mitigate oxidative stress, fortify the plant’s physical barriers, and bolster its ability to combat fungal pathogens. This increase in enzyme activity could enhance the antifungal properties of cherry tomatoes, preventing fungal infections and reducing decay caused by such pathogens.

### Effect of oligandrin treatment on expression of marker genes involved in SA and JA pathways

**Fig 4:**
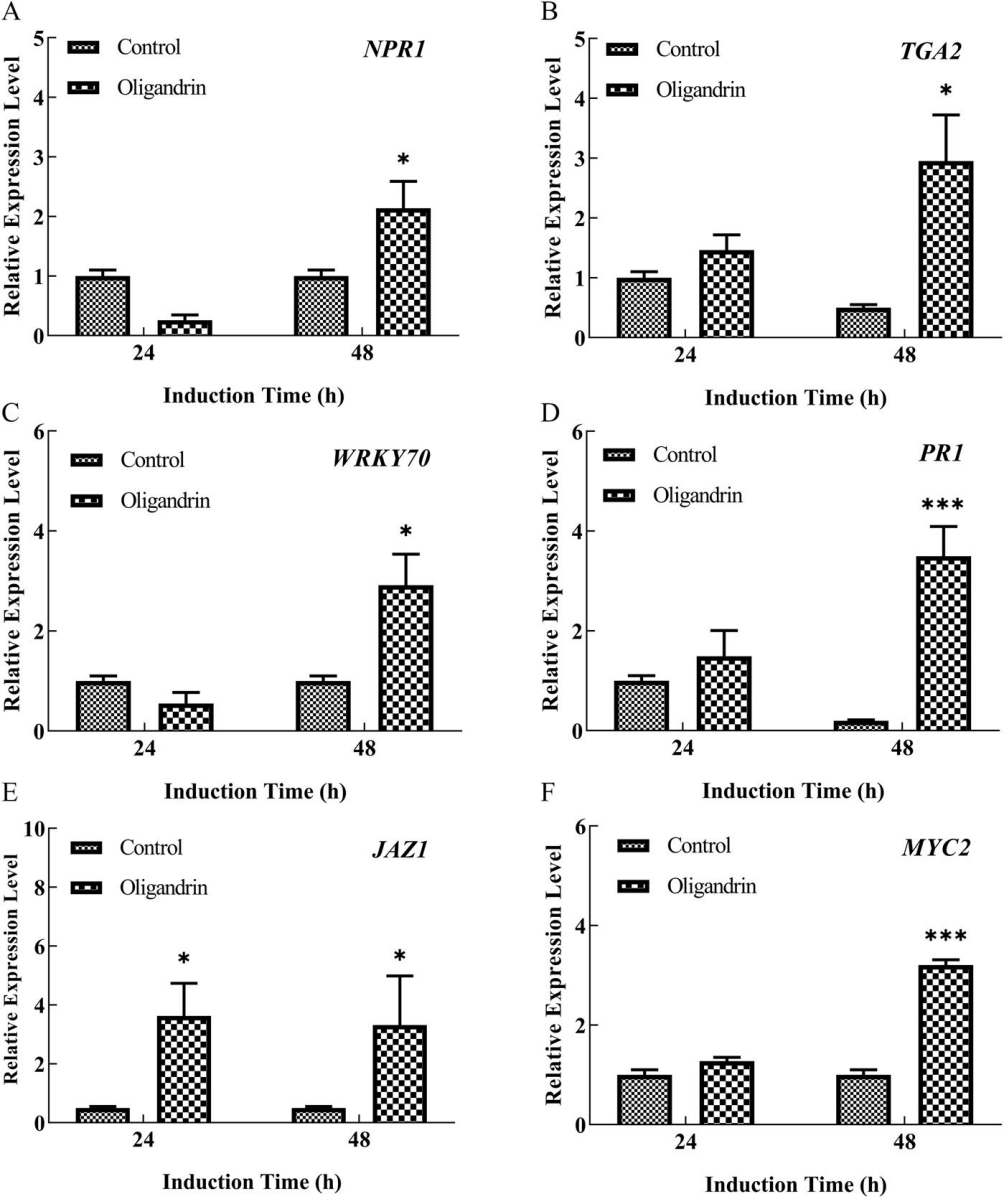
Effect of oligandrin on the expression of genes associated with salicylic acid (SA) signaling (**A –D**) and jasmonic acid (JA) signaling (**E and F**) in cherry tomatoes in response to *A. nees* infection. Gene expression levels are presented as relative values compared to the control at 24 and 48 h post-treatment. Error bars represent the standard error of the mean from three independent replicates. * P ≤ 0.05, ** P ≤ 0.01, and *** P ≤ 0.001 compared to the control at each time point.

The impact of oligandrin treatment on the expression of marker genes associated with SA and JA signaling pathways is shown in Fig. 4. The data reveal significant activation of the SA and JA signaling pathways in response to oligandrin treatment.

#### SA pathway activation

Oligandrin treatment resulted in a notable upregulation of Non-Expressor of Pathogenesis-Related Genes 1 (*NPR1*), a key regulator in the SA signaling pathway, with a 2.14-fold increase at 48 h compared to the control group (*P* < 0.05). This suggests that oligandrin induces the activation of SA-dependent defense mechanisms in cherry tomato. In line with the increase in *NPR1*, the expression of TGACG-Binding Factor 2 (*TGA2*), a downstream transcription factor in the SA pathway, also showed significant upregulation. At 24 and 48 h, the *TGA2* expression increased by 1.46- and 5.90-fold, respectively (*P* < 0.05), further supporting the activation of the SA pathway. These results are consistent with the known role of *TGA2* in mediating SA-induced defense responses in plants.

Additionally, the expression of *WRKY70*, a member of the WRKY transcription factor family that regulates SA-mediated defense, was markedly enhanced, showing a 2.9-fold increase at 48 h compared to the control (*P* < 0.05). Similarly, the downstream defense-related gene Pathogenesis-Related 1 (*PR1*), which is activated by SA signaling, exhibited a dramatic increase of 17.48-fold at 48 h (*P* < 0.05), underscoring the strong activation of the SA pathway in response to oligandrin. These findings collectively demonstrate that oligandrin treatment activates the SA signaling pathway, leading to the upregulation of key defense-related genes, which likely contributes to enhanced pathogen resistance and post-harvest preservation in cherry tomato.

#### JA pathway activation

Oligandrin treatment also influenced the JA signaling pathway. As shown in Fig. 4E and F, the expression of Jasmonate ZIM-domain protein 1 (*JAZ1*), a negative regulator of the JA pathway, was significantly upregulated, with 7.26- and 6.64-fold increases at 24 and 48 h, respectively (*P* < 0.05). Interestingly, the expression of *JAZ1* decreased by 8.54 % at 48 h compared to 24 h, suggesting a temporal modulation of the JA pathway. This suggests an initial suppression of the JA pathway, followed by subsequent activation, a phenomenon that may be indicative of a priming effect that bolsters the plant’s defense mechanisms over time.

Concurrently, the expression of *MYC2*, a central transcription factor in the JA pathway (24), showed a significant 3.21-fold increase at 48 h compared to the control (*P* < 0.001). This increase in *MYC2* expression supports the activation of the JA pathway in response to oligandrin treatment. The higher expression of *MYC2* at 48 h compared to 24 h indicates a sustained activation of the JA pathway following initial inhibition. The temporal expression patterns of *JAZ1* and *MYC2* indicate a complex regulatory mechanism in which the JA pathway is initially suppressed, followed by activation upon oligandrin treatment. This dynamic modulation of the JA signaling pathway enhances the antifungal activity of cherry tomatoes, thereby reducing fungal decay and inhibiting infections.

## DISCUSSION

In this study, oligandrin treatment resulted in the rapid and robust activation of both the SA and JA signaling pathways in harvested cherry tomatoes. This dual pathway activation was associated with enhanced disease resistance primarily through the upregulation of key defense-related genes (e.g., *NPR1*, *TGA2*, *WRKY70*, *PR1*, *JAZ1*, and *MYC2*) and an increase in defense-related enzyme activities (e.g., PPO, CAT, POD, and PAL). These findings presented here suggest that oligandrin, an active compound produced by *P. oligandrum*, may provide immediate protection against *A. nees* infection and prime the plant’s defense mechanisms. This dual activation of defense systems may offer a sustainable solution for post-harvest preservation and quality maintenance of berry-like crops, preventing decay and spoilage caused by the pathogenic fungus. In contrast to traditional chemical fungicides, which are often used for post-harvest disease control but come with limitations, such as toxicity, environmental impact, and pathogen resistance, oligandrin and *P. oligandrum* offer a more sustainable alternative.

Building on previous studies, our research underscores the pivotal role of the elicitin-like protein oligandrin in modulating harvested plant defense mechanisms. As highlighted in the literature, *P. oligandrum* induced both local and systemic resistance by activating key signaling pathways, particularly the ethylene (ET) and JA pathways (1). Oligandrin treatment has been shown to elevate the expression of various defense-related enzymes, including PPO, CAT, POD, and PAL, and promote the accumulation of phenolic and antifungal compounds (25). Our study extends these findings by demonstrating that oligandrin treatment in cherry tomatoes not only activates the JA pathway—evidenced by significant upregulation of *JAZ1* and *MYC2*—but also modulates the SA pathway, contributing to enhanced pathogen resistance and improved post-harvest preservation.

An important aspect of our study is the observed synergy between the SA and JA signaling pathways following oligandrin treatments. Interestingly, while *P. oligandrum* typically induces an ET- and JA-dependent defense response (26, 27), our findings reveal a more nuanced interaction between SA and JA pathways, suggesting that oligandrin can fine-tune the plant’s immune system, thereby promoting a broader and more robust defense mechanism. The interplay between SA and JA signaling pathways is well documented in plant immunity, with these pathways often working in tandem or in a coordinated manner to regulate defense responses (28, 29). The SA pathway, known for its pivotal role in SAR, involves key regulatory proteins like NPR1, TGA2, WRKY70, and PR1 (30). NPR1 not only interacts with TGA transcription factors to activate SA-responsive genes but also regulates the cross-talk between the SA and JA pathways to maintain immune balance (31). It is well-documented that SA and JA signaling often exhibit antagonistic interaction with NPR1, suppressing *MYC2* activation in the JA pathway (32). Despite this antagonism, oligandrin appeared to modulate both pathways simultaneously, overcoming this conflict to bolster the plant’s defense responses. This dual activation contrasts with previous reports where *P. oligandrum* was primarily linked to JA-induced resistance without clear involvement of the SA pathway (26, 27). Such a coordinated immune response may provide an efficient mechanism to resist pathogen attack while maintaining overall plant health, a feature that is critical in the context of post-harvest disease management. Additionally, transcriptome sequencing offers broad genetic data but may not pinpoint genes linked to oligandrin effects on resistance. The chosen methods here better capture crop resistance and observable responses.

**Fig 5:**
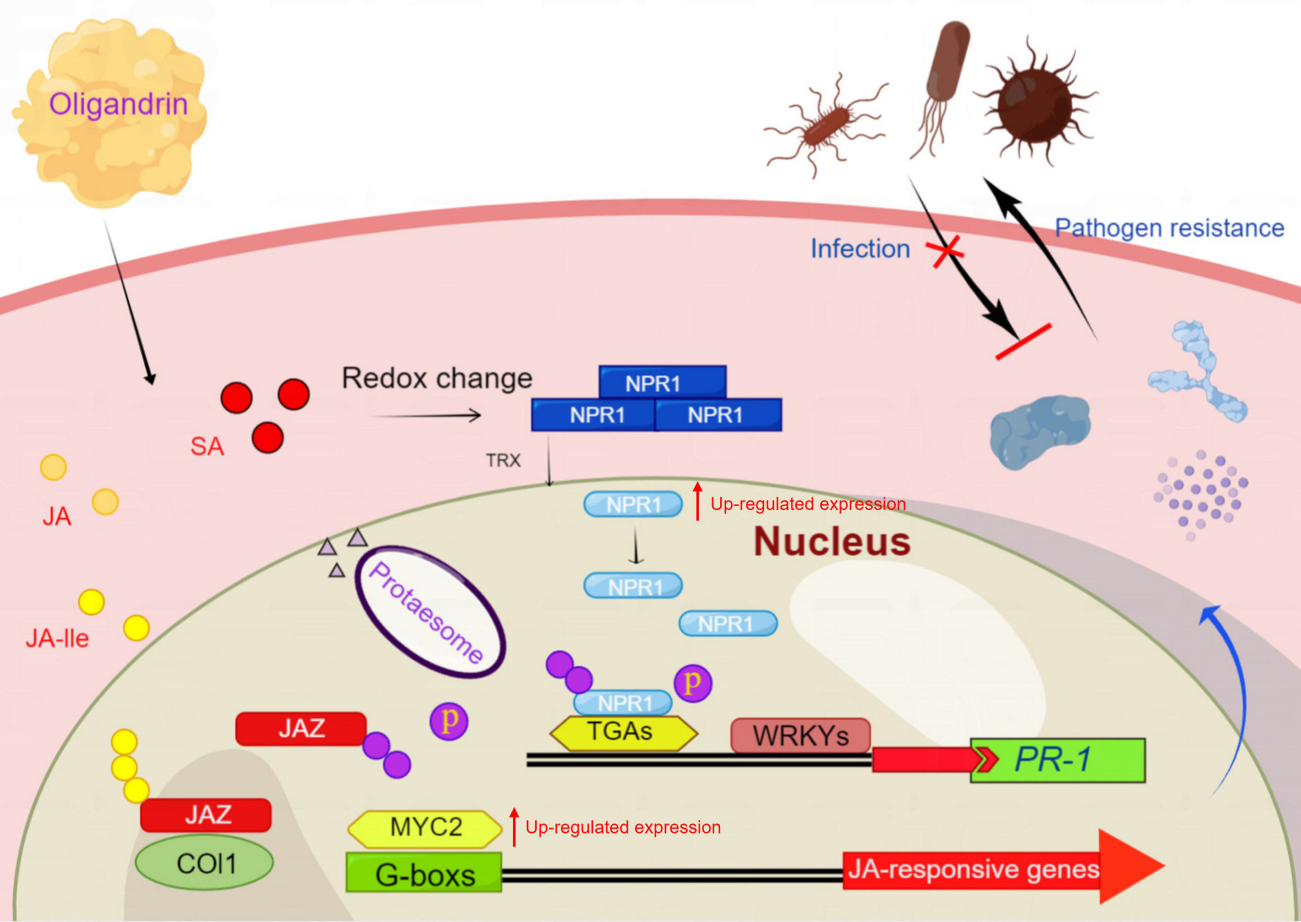
Schematic representation of the salicylic acid (SA) and jasmonic acid (JA) signaling pathways in cherry tomatoes following *A. nees* infection and oligandrin treatment. The figure illustrates the modulation of the SA signaling pathway upon oligandrin treatment, as well as the activation of the JA signaling pathway in cherry tomatoes infected with *A. nees* after treatment with oligandrin.

Previous research has demonstrated the potential of oligandrin in eliciting a defense response in plants, particularly in relation to *B. cinerea* in tomatoes, through activation of the ethylene-dependent signaling pathway (5). Additionally, oligandrin has been shown to induce HR in *Nicotiana benthamiana*, involving regulatory proteins, such as NbSGT1 and NbNPR1 (6). In vitro-purified PoEli8 from *P. oligandrum* induced strong innate immune responses and enhanced resistance to the oomycete pathogen *Phytophthora capsici* in *Solanaceae* plants, including *N. benthamiana*, tomato, and pepper (33). Our study uniquely highlights oligandrin’s ability to activate the SA signaling pathway, providing a deeper understanding of its role in plant fruit immunity. In our study, the significant upregulation of *NPR1*, *TGA2*, *WRKY70*, and *PR1* upon oligandrin treatment strongly supports the idea that oligandrin activates the SA pathway to prime the plant’s immune system, a process that may play a crucial role in enhancing post-harvest resistance to fungal pathogens. The modulation of the SA and JA pathways in response to oligandrin is visually represented in Fig. 5. This finding is consistent with previous research on other biocontrol agents and plant elicitors, such as flg22 from *Pseudomonas syringae* (34), which activates the plant immune system via the SA pathway. However, what sets our study apart is the specific and rapid induction of this immune response in harvested cherry tomatoes following oligandrin treatment, which has not been previously documented in this context.

Jasmonic acid exerts its effects by activating the expression of antibacterial proteins, and this process is tightly regulated by the binding of JA to the COII receptor and the subsequent release of the transcription factor MYC2 (35). Despite the known antagonism between SA and JA signaling, where NPR1 suppresses MYC2 activation (36), our study shows that oligandrin induces the expression of key JA genes, such as *JAZ1* and *MYC2*, suggesting that oligandrin may fine-tune both SA and JA pathways to enhance defense responses in cherry tomato against *A. nee*. This temporal regulation of *JAZ1* expression presented here is consistent with previous studies where *JAZ1* fluctuates in response to stress, allowing plants to fine-tune their immune responses (36). Alongside *JAZ1*, the expression of *MYC2* was significantly upregulated at 48 h (3.21-fold increase). The sustained activation of *MYC2* suggests that oligandrin treatment initiates a long-lasting defense response, which contributes to enhanced pathogen resistance (Fig. 5). These findings corroborate existing literature on the importance of MYC2 in regulating the plant immune response in response to JA signaling (36, 37).

By activating the plant’s own defense pathways, oligandrin and *P. oligandrum* offer a natural, eco-friendly alternative to chemical interventions for managing post-harvest diseases. Building on previous research and the present findings, we demonstrate that oligandrin can activate multiple signaling pathways, including ethylene (ET), JA, and SA, which together form a broad-spectrum defense against pathogens. The simultaneous activation of these pathways enhances the plant’s ability to resist a variety of pathogens, including *A. nees*, the causative agent of post-harvest fruit rot. In this study, the rapid induction of the SA pathway, as evidenced by the upregulation of *NPR*1, *TGA*2, and *WRKY*70, contributes to SAR, a defense mechanism that provides protection against a wide range of pathogens (31). Additionally, the increased expression of defense-related enzymes, such as PPO, CAT, POD, and PAL, indicates that oligandrin enhances pathogen resistance and potentially prolongs fruit shelf life. These enzymes play critical roles in oxidative stress responses, phenolic biosynthesis, and cell wall fortification, which collectively help inhibit pathogen entry (37). This finding underscores the potential of oligandrin to improve post-harvest fruit quality by activating the plant’s intrinsic immune system. From a molecular perspective, it highlights *P. oligandrum* as a promising biopesticide for global use in post-harvest preservation and antifungal applications. Furthermore, the observed temporal regulation of *JAZ1* and *MYC2* expressions—initial suppression, followed by sustained activation—mirrors immune priming dynamics seen in other plant-pathogen interactions involving *P. oligandrum* (38). Importantly, oligandrin treatment significantly reduced disease symptoms, such as black spot and decay, during storage without inhibiting *A. nees* spore germination, further confirming its potential for post-harvest disease management. This suggests that oligandrin effectively primes the plant’s defense system, enhancing resistance and extending fruit shelf life.

The findings of this study have important implications for post-harvest disease management, particularly for crops like cherry tomatoes, where preserving fruit quality during storage remains a significant challenge. By activating both the SA and JA pathways, oligandrin not only enhances pathogen resistance but also helps extend shelf life and reduce spoilage. This dual activation mechanism, coupled with the sustained immune response observed, positions oligandrin and its producer, *P. oligandrum*, as a promising tool for post-harvest preservation and quality maintenance. These results suggest the potential for a shift toward more sustainable agricultural practices. While the efficacy of oligandrin at the laboratory scale is well established, its performance under varying storage conditions (e.g., temperature fluctuations) still requires further validation. Additionally, the identity of the receptor(s) for oligandrin remains unknown, which limits the ability to fully optimize its mechanism of action. Future studies could utilize *NPR1/MYC2* gene-edited lines to explore the contributions of specific pathways and test oligandrin against a broader range of post-harvest pathogens. Overall, these findings underscore *P. oligandrum* as a promising biopesticide for post-harvest preservation, offering an eco-friendly alternative to chemical treatments for mold prevention and improved fruit quality.

### Conclusions

This study provides novel evidence that oligandrin activates both the SA and JA signaling pathways in cherry tomatoes, thereby enhancing resistance to *A. nees*. This dual activation leads to the upregulation of key defense-related genes and an increase in defense-related enzyme activities, strengthening the plant’s immune response. Notably, oligandrin significantly reduces disease incidence caused by *A. nees*, highlighting the potential of this protein and its producer *P. oligandrum* as a sustainable alternative to chemical pesticides for post-harvest preservation. By modulating SA and JA pathways and activating defense enzymes, oligandrin not only bolsters plant immunity but also helps maintain fruit quality during storage. Given that *P. oligandrum*, the producer of oligandrin, is already established as a biopesticide in Europe and America, these findings offer an environmentally friendly solution to crop protection and food safety. This research opens new avenues for post-harvest disease management, supporting that *P. oligandrum* can be used in post-harvest preservation of cherry tomato-like fresh crops. However, further studies are needed to refine its application, particularly under real-world conditions. Continued research is crucial to fully harness the potential of *P. oligandrum* and oligandrin as a reliable, sustainable tool in agricultural and post-harvest management, advancing biocontrol strategies and promoting safer, more sustainable food production systems.

### Highlights

- Oligandrin reduces post-harvest disease in cherry tomatoes without inhibiting spore germination.
- Oligandrin activates SA and JA pathways, upregulating *NPR1*, *TGA2*, *PR1*, and *MYC2* genes.
- Enhanced activities of defense enzymes correlate with oligandrin-induced systemic resistance.
- Oligandrin primes SA defense and modulates JA signaling, enhancing post-harvest preservation.
- Study provides insights into oligandrin's role in sustainable post-harvest management.
- Findings support broader application of *Pythium oligandrum* in post-harvest preservation.

## Data Availability

All relevant data are included in the article and its supplemental material files at https://github.com/jiahaoSun-1205/AEM00421-25R1_Supplemental_material/blob/main/AEM00421-25R1_Supplemental_material.docx.
